# Bitter Taste Perception in BaYaka Hunter‐Gatherers

**DOI:** 10.1002/ajhb.70218

**Published:** 2026-02-18

**Authors:** Sarai Keestra, Inez Derkx, Edmond Sylvestre Miabangana, Gaurav Sikka, Nikhil Chaudhary, Gul Deniz Salali

**Affiliations:** ^1^ Department of Epidemiology and Data Science, Amsterdam UMC, University of Amsterdam Amsterdam the Netherlands; ^2^ Paediatric Endocrinology, Emma Kinderziekenhuis, Amsterdam UMC University of Amsterdam Amsterdam the Netherlands; ^3^ Department of Anthropology University of Zürich Zürich Switzerland; ^4^ Agence Nationale de Valorisation des Résultats de la Recherche et de l'innovation (ANVRI), Cité Scientifique de Brazzaville Brazzaville Republic of the Congo; ^5^ Herbier National du Congo (IEC), Cité Scientifique de Brazzaville Brazzaville Republic of the Congo; ^6^ NHS Greater London London UK; ^7^ Leverhulme Centre for Human Evolutionary Studies, Department of Archaeology University of Cambridge Cambridge UK; ^8^ Department of Anthropology University College London London UK

**Keywords:** BaYaka hunter‐gatherers, bitter taste, dietary transition, hunter‐gatherers, plant ecology, PTC, thiourea, traditional medicine

## Abstract

**Objectives:**

This study examined variation in bitter taste perception among BaYaka hunter‐gatherers from the Republic of Congo, comparing individuals from the same population that were born and grew up in a forest ecology to those from a logging town.

**Methods:**

Bitter‐tasting phenotype was assessed in 112 BaYaka individuals using a paper‐strip taste‐detection task with single‐concentration strips of phenylthiocarbamide (PTC) and thiourea (thiocarbamide). Participants were grouped by the place where they were born and grew up: forest camps or the town. Logistic regression was used to test associations between location, sex, age, and bitter taste perception.

**Results:**

Town‐born individuals were more likely to perceive both compounds as bitter than forest‐born individuals (PTC: OR = 3.93, 95% CI: 1.75–9.17, *p* < 0.01; thiourea: OR = 4.44, 95% CI: 1.97–10.42, *p* < 0.01). No significant associations were found between bitter taste perception and sex or age.

**Conclusion:**

Bitter‐tasting phenotype differed among BaYaka individuals, with higher proportions of bitter tasters among those born and raised in town compared to those from forest camps. These results suggest that early‐life ecological context may contribute towards variation in bitter taste perception, which we hypothesize might be due to differences in exposure to bitter wild plant compounds.

## 1 Introduction

Bitter taste perception is a sensory adaptation enabling detection and avoidance of toxic plant compounds, particularly secondary metabolites (Wooding 2006; Wooding et al. 2021). In humans, this ability is mediated by 25 TAS2R receptors responding to structurally diverse ligands (Wooding et al. 2021). Many bitter compounds act as plants' chemical defenses but can be harmful at high doses (Muzzaffar et al. 2019). For example, glucosinolates in cruciferous vegetables and cyanogenic glycosides in cassava can be metabolized into goitrogens, disrupting iodine uptake and thyroid function, and give these foods a distinct bitter taste (Muzzaffar et al. 2019). Not all compounds activating TAS2R receptors are toxic; some, such as caffeine in coffee and polyphenols in green tea, are harmless in small doses (Nissim et al. 2017), whereas others such as acetylsalicylic acid from willow bark have medicinal properties (Meyerhof et al. 2009). The omnivore's dilemma is judging when bitter foods are toxic or beneficial, with sensitivity shaped by evolutionary pressure to balance toxin avoidance and medicinal use (Balick and Cox 2020; Wooding et al. 2021).

Bitter‐tasting phenotype varies widely across human populations. Although TAS2R bitter receptor variation explains part of this, studies have not identified consistent associations between genotype and diet or geography (Campbell et al. 2012). Phenotyping studies in small‐scale societies for the inability to taste the synthetic bitter compound phenylthiocarbamide (PTC) show substantial variation, with non‐taster frequencies ranging from ~2% to 3% in Amazonian groups to over 60% in South Asian horticulturalists (Guo and Reed 2001). Even neighboring populations can differ: Baka hunter‐gatherers in Cameroon have higher detection thresholds for quinine, an alkaloid derived from *Cinchona* bark and used in antimalarial treatment, than neighboring Bantu farmers, even though Baka diets include many more wild plant species (Sjöstrand et al. 2020). Sjöstrand et al. propose that environmental factors, including the broader range of plant foods typically consumed by hunter‐gatherers, may help explain phenotypic differences in bitter‐taste sensitivity between populations. This raises the question whether variation within a single population could also arise from differences in habitual plant use.

The Mbendjele BaYaka are hunter‐gatherers in the northeastern forest of Congo‐Brazzaville and related to the Baka in Cameroon. They use wild plants for treating infections and other illnesses (Salali et al. 2016). However, with increasing integration in the market economy, their engagement with wild plants varies (Salali 2017; Salali et al. 2020). Town‐born BaYaka report knowing and using fewer medicinal plants compared to those in forest camps (Salali et al. 2020). Forest‐born individuals rely more on traditional medicine, regularly consuming bitter barks, leaves, and roots; town‐born BaYaka often favor Western medicine (Salali et al. 2020). Living in town offers greater access to cultivated food, such as cassava (Keestra et al. 2024). As this is the first generation in which some BaYaka have been raised entirely in town, we tested whether this shift in early‐life environment corresponds to differences in bitter‐tasting phenotype using a single‐concentration task with PTC and thiourea.

## 2 Materials and Methods

We included 112 BaYaka (57 women, 55 men) from the Republic of Congo's Ndoki forest July–August 2018. Thirty‐nine participants were born and raised in a logging town, where they engage more in wage labor, trade with farmers, and have access to cultivated food from the market (Salali and Migliano 2015). Seventy‐four came from forest camps > 3 h from town. Ethical approval was granted by University College London (13 121/001) and the Republic of Congo's Ministry of Scientific Research; participants provided informed consent. Participants tasted three strips in a fixed order: a control strip, then PTC (20 μg/strip) and thiourea (60 μg/strip); (Eisco Precision Laboratories). For each strip, a trained BaYaka assistant asked the open‐ended question of what the paper tasted like, with responses translated into French. We classified participants as ‘tasters’ if describing PTC or thiourea as bitter compared to control; those reporting similarity to the control (e.g., tasteless, slightly sweet, salty) were classified as ‘non‐tasters’. Logistic regression tested associations between location and bitter‐tasting phenotype, adjusting for sex; sensitivity analyses assessed age and phenotype overlap. Analyses used R v4.2.0.

## 3 Results

**FIGURE 1:**
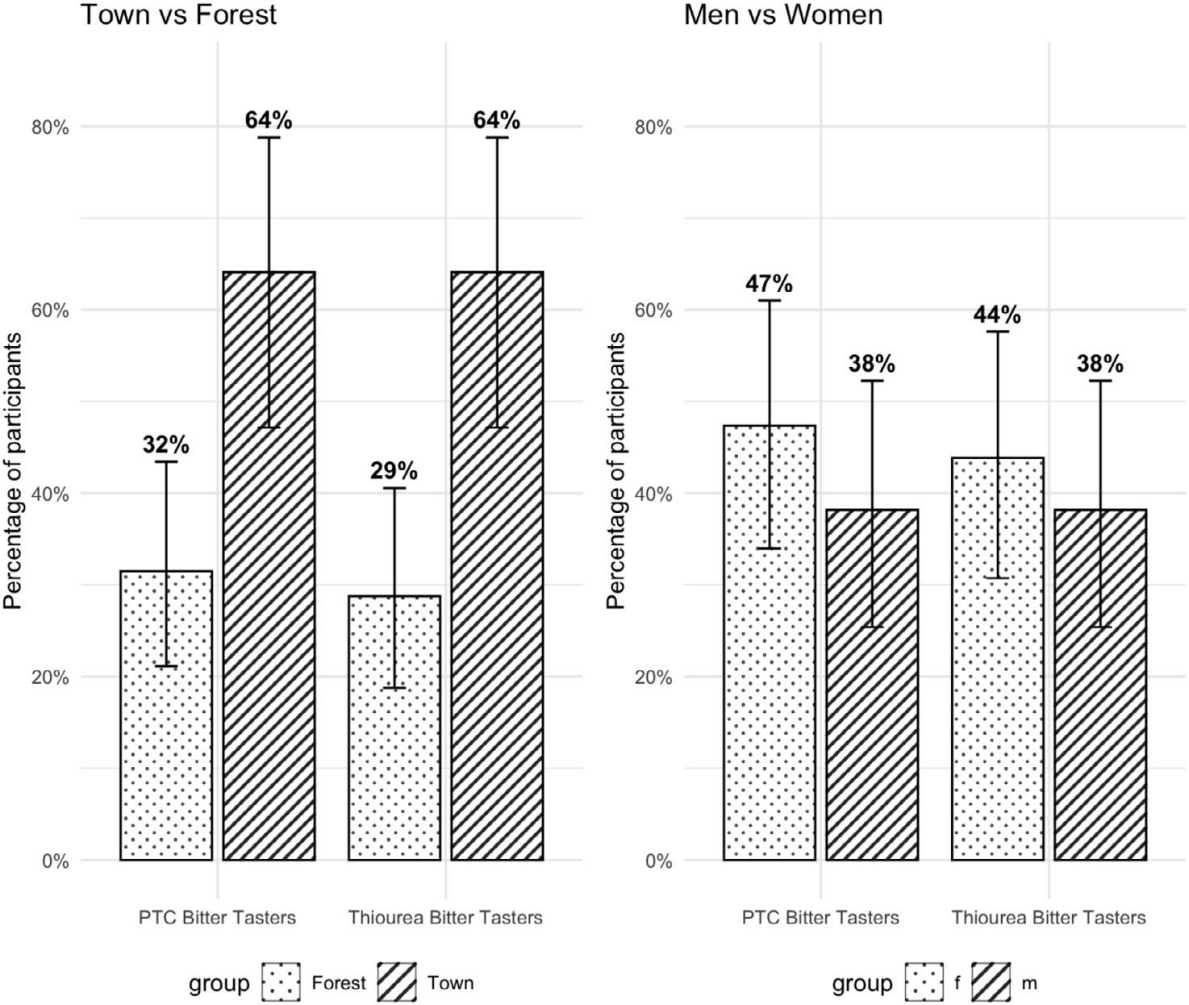
Percentage of PTC and thiourea bitter tasters in BaYaka hunter‐gatherers by location (forest‐born BaYaka *n* = 74 and town‐born BaYaka *n* = 39) and sex (women *n* = 57 and men *n* = 55).

Of the 112 BaYaka, 43% (48/112) were PTC tasters, 53% (59/112) non‐tasters; 4.5% (5/112) described it as hot/spicy. For thiourea, 41% (46/112) were tasters, 57% (64/112) were non‐tasters, and 1.8% (2/112) reporting hot/spicy. Bitter‐tasting phenotype was more frequent in town‐born than forest‐born BaYaka (64% vs. 32% for PTC and 64% vs. 29% for thiourea) (Figure 1). Being in town increased odds of being a taster (PTC: OR = 3.93, *p* < 0.01; thiourea: OR = 4.44, *p* < 0.01) (Table 1). Twenty‐one percent (24/112) were tasters for both compounds (Table S1). No significant differences in bitter taste perception were found based on sex or age (Table 1; Table S2).

**TABLE 1:** Logistic regression models for PTC and Thiourea bitter‐tasting phenotype in BaYaka hunter‐gatherers by location and sex.

	Odds ratio	95% confidence interval	*p*
PTC			
Town vs. forest	3.93	1.75–9.17	0.001
Men vs. women	0.66	0.30–1.46	0.311
Thiourea			
Town vs. forest	4.44	1.97–10.42	0.0004
Men vs. women	0.77	0.34–1.73	0.530

## 4 Discussion

Within BaYaka hunter‐gatherers, there was significant variation in bitter‐tasting phenotype depending on where participants were from. BaYaka in town were more likely to perceive PTC and thiourea as bitter compared to BaYaka residing in the forest. The proportion of tasters in town (64%) closely matches that of Baka in Cameroon (63%) (Sjöstrand et al. 2020), while only a third of BaYaka in forest camps described the strips as bitter. This places BaYaka in the forest at the higher end of global PTC non‐taster frequencies. Table S3 summarizes published percentages across small‐scale populations (Guo and Reed 2001), which span wide ranges within each subsistence mode.

Evolutionary explanations suggest that tasting and non‐tasting phenotypes can have context‐dependent advantages. Non‐tasters may be more open to a broader range of plant‐based foods or able to ingest medicinal plants more readily (Wooding et al. 2006) (Salali et al. 2016). In non‐human primates, reduced bitterness sensitivity has evolved as a dietary adaptation to leaf‐eating (Wooding et al. 2021). Our findings align with Sjöstrand et al. (2020), who found lower tasting frequency among Baka than in neighboring Bantu farmers. Many plants used by BaYaka in the forest contain bitter compounds like alkaloids, saponins, and tannins, and are used to treat digestive and respiratory disorders (Table S4) (Salali et al. 2016). In high‐pathogen environments, tolerance to bitter compounds might confer benefits in low doses, as these compounds often possess anti‐parasitic or antimicrobial properties (Sjöstrand et al. 2020; Wooding et al. 2006, 2021). This offers one explanation for our results, though it requires further investigation.

Reduced bitterness perception may also entail vulnerabilities in changing environments as the BaYaka undergo a transition towards increased market integration (Knight et al. 2021; Salali and Migliano 2015). Individuals with lower bitter sensitivity experience fewer deterrents to alcohol and consume alcoholic beverages more frequently (Duffy et al. 2004); among BaYaka, where hazardous alcohol use is increasing (Knight et al. 2021), reduced aversion to bitter tastes may make alcohol use more likely. As cassava becomes more widely cultivated and traded in the region, lower bitter sensitivity could weaken sensory cues that signal toxicity of inadequate processing of higher‐cyanide varieties, although these varieties are not predominant in northern Congo (Keestra et al. 2024; Muzzaffar et al. 2019; Wooding et al. 2021). Sensory ecology in relation to these shifting dietary risks merits further study.

Our study had limitations. The small sample size reflects the tight‐knit nature of BaYaka communities, with < 60 individuals in many camps (Knight et al. 2021; Salali 2017). As chronological age is not routinely recorded in this population, we were unable to examine associations between age and bitter taste perception with accuracy (Table S3). Our single‐concentration task captures only one aspect of bitterness perception and may overestimate non‐tasters, though this is unlikely to affect between‐group differences. To address potential concerns about genetic differentiation, we note that the settlement in town was established only in the past three decades. BaYaka mobility is high, and individuals frequently move between town and forest settlements to visit or reside with relatives. Thus, genealogical networks span multiple locations, and the number of generations separating ‘town‐born’ and ‘forest‐born’ individuals is too low to lead to meaningful genetic divergence. Moreover, admixture with neighboring Bantu farmers remains extremely limited. Our genealogical records for all individuals in town show only two cases of BaYaka–Bantu intermarriage among 305 individuals residing there. These factors indicate that town and forest BaYaka constitute a single, fluid population with minimal external gene flow. Although movement between forest and town settlements is common, this typically involves temporary visits from forest to town for family visits or market‐related activities. To avoid conflating temporary mobility with long‐term residence, our town‐based sample included only individuals who were born and raised in the town settlement rather than in forest camps. The differences we report in bitter taste perception are therefore unlikely to reflect underlying genetic structure and instead point towards environmental or developmental influences. Future studies should include different concentrations of bitter compounds, assess presence of bitter compounds in foraged plants, and examine taste terminology in the BaYaka language.

## 5 Conclusion

BaYaka residing in town more often had a bitter‐tasting phenotype than those living in the forest, where the prevalence of bitter tasting was exceptionally low, indicating an environmental component in bitter taste perception. These findings require cautious interpretation due to sample size, single‐concentration design, and lack of genetic data. Research combining taste‐threshold tests, TAS2R genotyping, and detailed records of dietary and medicinal plant exposure will be essential to clarify the environmental and genetic contributions to bitter taste variation.

## Author Contributions

G.D.S. conceived the project; S.K., G.D.S., N.C., G.S., and I.D., collected the data; E.S.M. helped with the literature review on bitter compounds found in medicinal plants; S.K. conducted the analyses and wrote the manuscript under the supervision of G.D.S.; all authors reviewed and approved the final manuscript.

## Funding

This project was funded by the British Academy research grant (SRG\171409) to G.D.S.

## Conflicts of Interest

The authors declare no conflicts of interest.

## Supporting information


**Data S1:** ajhb70218‐sup‐0001‐Supplementary1.docx.

**Data S2:** ajhb70218‐sup‐0002‐Supplementary2.docx.

**Data S3:** ajhb70218‐sup‐0003‐Supplementary3.docx.

**Data S4:** ajhb70218‐sup‐0004‐Supplementary4.docx.

## Data Availability

The data that support the findings of this study are openly available in Open Science Framework at https://osf.io/6t4vd/overview, reference number 6t4vd.
