# Supplementary material for: Bitter Taste Perception in BaYaka Hunter‐Gatherers
Source: Am J Hum Biol. 2026 Feb 18;38(2):e70218. doi: 10.1002/ajhb.70218 (PMC12916250; doi:10.1002/ajhb.70218)
Supplement: Supplementary file 1 — Data S1: ajhb70218‐sup‐0001‐Supplementary1.docx. [file AJHB-38-e70218-s004.docx]

Supplementary 1

Overlap between PTC and thiourea bitter tasting

These are the results for the analysis of bitter taste perception in BaYaka hunter-gatherers examining the overlap between PTC and thiourea bitter tasting. We did not find that being able to perceive one compound significantly predicted tasting the other (p = 0.60; OR = 1.26; 95% CI: 0.53–2.92), after adjusting for sex and birthplace. We recount here who of the bitter tasters thought that the other compound was also bitter. Of the PTC bitter tasters, 25 found thiourea also bitter, 22 did not and 1 found it hot. Of the thiourea bitter tasters, 24 found PTC also bitter, 20 did not and 2 found it hot. To conclude, there were 24 participants who experienced both compounds as bitter. In the regression below, we examined whether thiourea tasting predicted PTC tasting controlling for sex and birthplace. The only significant variable was the birthplace; perceiving thiourea as bitter did not predict perceiving PTC bitter (Table 1).

Table 1. Odds of PTC tasting with respect to thiourea tasting, sex and birthplace

|  | Odds ratio | 95% Confidence Interval | P-value |
| --- | --- | --- | --- |
| Thiourea tasting | 1.26 | 0.53-2.92 | 0.60 |
| Men vs women | 0.67 | 0.33-1.48 | 0.33 |
| Town vs forest | 3.63 | 1.54-8.89 | 0.004 |
