# Supplementary material for: Bitter Taste Perception in BaYaka Hunter‐Gatherers
Source: Am J Hum Biol. 2026 Feb 18;38(2):e70218. doi: 10.1002/ajhb.70218 (PMC12916250; doi:10.1002/ajhb.70218)
Supplement: Supplementary file 2 — Data S2: ajhb70218‐sup‐0002‐Supplementary2.docx. [file AJHB-38-e70218-s002.docx]

Supplementary 2

Age is not significantly associated with bitter taste perception

Below are the results for the analysis of bitter taste perception in BaYaka hunter-gatherers when age is included in the regression model. Note that not for all participants age was available, as BaYaka do not record their age in years such as in the Western cultural tradition. We had age estimates for 40 participants from the forest camps only. Those age estimates were calculated based on the method described in Diekmann et al 2017.

We conducted a regression analysis with this subset of the data where we had age estimates. We did not find a significant association between age and bitter taste perception in this population (Table 1), but some indication that age could matter for perceiving thiourea as bitter, deserving further investigation.

Table 1. Odds of PTC and Thiourea tasting with respect to age

|  | Odds ratio | 95% Confidence Interval | P-value |
| --- | --- | --- | --- |
| PTC | | |  |
| Age (years) | 0.99 | 0.95-1.03 | 0.61 |
| Thiourea | | |  |
| Age (years) | 0.96 | 0.91-1.00 | 0.06 |

Next, we ran regression models including both age and sex. The results are shown in the table below. There was no significant association of age and bitter taste perception, controlling for sex, however the association between sex and age and thiourea bitter tasting phenotype deserves further investigation.

Table 2. Odds of PTC and Thiourea tasting with respect to age and sex

|  | Odds ratio | 95% Confidence Interval | P-value |
| --- | --- | --- | --- |
| PTC | | |  |
| Sex | 0.67 | 0.20-2.82 | 0.56 |
| Age | 0.99 | 0.95-1.03 | 0.68 |
| Thiourea | | |  |
| Sex | 0.30 | 0.07-1.15 | 0.09 |
| Age | 0.96 | 0.91-1.00 | 0.08 |

References:

Diekmann, Y., Smith, D., Gerbault, P., Dyble, M., Page, A. E., Chaudhary, N., Migliano, A. B., et al. (2017). Accurate age estimation in small-scale societies. *Proceedings of the National Academy of Sciences of the United States of America*, *114*(31).
