# Supplementary material for: Bitter Taste Perception in BaYaka Hunter‐Gatherers
Source: Am J Hum Biol. 2026 Feb 18;38(2):e70218. doi: 10.1002/ajhb.70218 (PMC12916250; doi:10.1002/ajhb.70218)
Supplement: Supplementary file 3 — Data S3: ajhb70218‐sup‐0003‐Supplementary3.docx. [file AJHB-38-e70218-s001.docx]

Supplementary 3

Non-taster percentage for PTC in small-scale societies based on Guo & Reed (2001) with subsistence mode added

| Population | Country or Region | Subsistence | n | Non taster percentage % | Reference |
| --- | --- | --- | --- | --- | --- |
| Hadza | Tanzania | Hunter-gatherers | 118 | 23.7 | Barnicot & Woodburn 1975 |
| San | Southern Africa | Hunter-gatherers | 85 | 7.1 | Jenkins 1965 |
| Australian Aboriginal | Australia | Hunter-gatherers | 152 | 49.3 | Simmons et al. 1954a |
| Highland Papuans from Enarotali (Paniai Lakes region) | New Guinea | Horticulturalists | 178 | 36 | Graydon et al. 1958 |
| Jivaro (Shuar) | Ecuador Amazon | Horticulturalists (with hunting/fishing) | 327 | 2.1 | Sunderland & Ryman 1968 |
| Tucano | Brazil Amazon | Horticulturalists (with fishing) | 128 | 6.3 | Montenegro 1964 |
| Kaingang | Brazil | Horticulturalists | 77 | 2.6 | Kalmus 1957; Fernandes 1957 |
| Carajas | Brazil | Horticulturalists (with fishing) | 86 | 0 | Kalmus 1957; Junqueira 1957 |
| Highland Quechua | Peru (Andean highlands) | Agropastoralists | 319 | 3.1 | Frisancho et al. 1977 |
| Lowland Quechua | Peru (Amazonian foothills) | Horticulturalists (with foraging/fishing) | 672 | 7.1 | Frisancho et al. 1977 |
| Southern Peruvian Quechua | Peru (Southern Andes) | Agropastoralists | 522 | 2.9 | Garruto et al. 1975 |
| Aeta | Philippines | Hunter-gatherers | 73 | 13.7 | Pascasio et al. 1974 |
| Northern Pahira | India | Forager-horticulturalists | 206 | 41.6 | Basu et al. 1966 |
| Southern Pahira | India | Horticulturalists | 671 | 65.7 | Basu et al. 1966 |
| Kondhs (Orissa) | India | Horticulturalists | 51 | 64.7 | Tripathy 1966 |
| Pardhans | India | Horticulturalists | 140 | 62.9 | Goud & Rao 1979a |

Above table includes small-scale forager, hunter-gatherer, horticulturalist and agropastoralist societies from the overview provided by Guo & Reed (2001) in Table 1 "Table 1. Worldwide population variation in nontaster frequency." For the full table and bibliography we refer to

Guo, S. W., & Reed, D. R. (2001). The genetics of phenylthiocarbamide perception. *Annals of Human Biology*, *28*(2), 111–142. https://doi.org/10.1080/03014460151056310

**Hadza**
Barnicot, N.A. & Woodburn, J., 1975. Colour-blindness and sensitivity to PTC in Hadza. Annals of Human Biology, 2, pp.61–68.

**San (Southern Africa)**
Jenkins, T., 1965. Ability to taste phenylthiocarbamide among Kalahari Bushmen and Southern Bantu. Human Biology, 37, pp.371–374.

**Australian Aboriginal**
Simmons, R.T., Graydon, J.J. & Semple, N.M., 1954. A blood group genetical survey in Australian Aborigines. American Journal of Physical Anthropology, 12, pp.599–606.

**Highland Papuans (Enarotali, Paniai Lakes region)**
Graydon, J.J. et al., 1958. Blood groups in Pygmies of the Wissellakes in Netherlands New Guinea. American Journal of Physical Anthropology, 16, pp.149–171.

**Jívaro (Shuar)**
Sunderland, E. & Ryman, R., 1968. P.T.C. thresholds, blood factors, colour vision and fingerprints of Jivaro Indians in Eastern Ecuador. American Journal of Physical Anthropology, 28, pp.339–344.

**Tucano (Brazil)**
Montenegro, L., 1964. P.T.C. tasting among Tucano Indians. Annals of Human Genetics, 28, pp.185–187.

**Kaingang (Brazil)**
Fernandes, J.L. et al., 1957. P.T.C. thresholds, colour vision and blood factors of Brazilian Indians I. Kaingangs. American Journal of Human Genetics, 22, pp.16–21.

**Carajás (Brazil)**
Junqueira, P.C., Kalmus, H. & Wishart, P., 1957. P.T.C. thresholds, colour vision and blood factors of Brazilian Indians II. Carajas. Annals of Human Genetics, 22, pp.22–25.

**Highland Quechua (Peruvian Andes)**
Garruto, R. et al., 1975. Phenotypic variation in ABO and Rh blood groups, PTC tasting ability, and lingual rotation among Southern Peruvian Quechua Indians. Human Biology, 47, pp.193–199.

**Lowland Quechua (Amazonian foothills)**
Frisancho, A.R. et al., 1977. Taste sensitivity to phenylthiourea (PTC), tongue rolling, and hand clasping among Peruvian and other Native American populations. Human Biology, 49, pp.155–163.

**Southern Peruvian Quechua**
Garruto, R. et al., 1975. Phenotypic variation in ABO and Rh blood groups, ﻿PTC tasting ability, and lingual rotation among Southern Peruvian Quechua Indians. Human Biology, 47, pp.193–199.

**Aeta (Philippines)**
Pascasio, F.M. et al., 1974. Genetic marker systems in Philippine negritos. Birth Defects: Original Article Series, 10, pp.220–225.

**Northern and Southern Pahira (India)**
Basu, A. et al., 1966. Anthropogenetic investigations in the Dalma and the Ajodhya Hills and their neighborhood. Science and Culture, 32, pp.273–275.

**Kondhs, Orissa (India)**
Tripathy, K.C., 1966. Taste, mid-digital hair and occipital hair whorls of Kondhs, Orissa. Adibasi, 7, pp.29–36.

**Pardhans (India)**
Goud, J.D. & Rao, P.R., 1979. Colour-blindness and taste sensitivity to phenylthiocarbamide in tribal populations of Andhra Pradesh. Journal of Indian Anthropological Society, 14, pp.269–274.
