# Supplementary material for: Bitter Taste Perception in BaYaka Hunter‐Gatherers
Source: Am J Hum Biol. 2026 Feb 18;38(2):e70218. doi: 10.1002/ajhb.70218 (PMC12916250; doi:10.1002/ajhb.70218)
Supplement: Supplementary file 4 — Data S4: ajhb70218‐sup‐0004‐Supplementary4.docx. [file AJHB-38-e70218-s003.docx]

Supplementary 4

Plants used for medical purposes by the BaYaka and the bitter compounds they contain

| Latin name | Bitter compounds |
| --- | --- |
| *﻿* [Oncoba welwitschi*i Oliv.*](https://www.ville-ge.ch/musinfo/bd/cjb/africa/details.php?langue=fr&id=16721) (Salicaceae) | The bitter taste of the plant is due to the presence in the seeds of chaulmoogric acid, formerly used in the treatment of leprosy [1]. Additionally, the presence of hydrocyanic acid has been detected [2]. |
| *Rauvolfia vomitoria Afzel (Apocynaceae)* | The roots contain many bitter tasting alkaloids, the most active being reserpine and rescinnamine. Others include reserpoxidine, seredine, ajmaline, alstonine, isoajmaline, isoreserpiline, raumatorine, rauvomitine, reserpiline, serpagine, vomalidine, yohimbine, and α-yohimbine [3]. |
| *Alstonia boonei De Wild. (Apocynaceae)* | The bark of the trunk is rich in alkaloids, quinones, terpenoids and coumarins [4]. |
| *﻿**Croton haumaniamus* J. Léonard (Euphorbiaceae) | *Croton* trees are high in active alkaloids [5]. The bitter bark of the related *Croton eluteria* is used in the traditional medicine of northern South America as well [5]. |
| *﻿Ricinodendron heudelotii* (Baill.) Pierre ex Heckel (Euphorbiaceae) | The seeds from the fruit of the *Ricinodendron* have a spicy/peppery taste[6]. |
| *﻿Musanga cecropioides* Rbr. (Urticaceae)  [*Piptadeniastrum africanum* (Hook. f.) Brenan](https://www.ville-ge.ch/musinfo/bd/cjb/africa/details.php?langue=fr&id=70010) (Fabaceae) | Extract of this nettle type plant contains alkaloids terpens and saponins which have a bitter and acrid taste [7]. There is a presence of saponins and tannins in trunk and root bark [2]. |
| [*Pentaclethra macrophylla* Benth.](https://www.ville-ge.ch/musinfo/bd/cjb/africa/details.php?langue=fr&id=70031) (Fabaceae) | The stem bark contains high concentrations of bitter saponins, tanins, and alkaloids [8,9]. The root contains saponins, numerous polyphenols (tannins) and steroids [10]. |
| *﻿Zanthoxylum tessmannii*  See under:  [Zanthoxylum gilletii (De Wild.) P.G. Waterman](https://www.ville-ge.ch/musinfo/bd/cjb/africa/details.php?langue=fr&id=89769) (Rutaceae) | Bitter tasting alkaloidal (peroxysimulenoline, sanguinarine, xanthoplanine, fagarine I and norchelerythrine), monoterpenes (myrcene, limonene, and camphene) compounds have been isolated from *Zanthoxylum* species [11]. The phytochemical study by TLC of a coumarin extract of Zanthoxylum gilletii leaves revealed in addition to coumarins, the presence of flavonoids and anthracene derivatives [12]. |
| *Microdesmis puberula* Hook. f. exPlanch*.*  (Pandaceae) | Alkaloid traces found in stems and roots of this plant [13,14], which are typically perceived as bitter [15]. |
| *Irvingia gabonensis (Aubry-LeComte ex O'Rorke) Baill. (Irvingiaceae)* | Presence of saponins and tannins has been detected in the barks and roots of other Irvingiaceae from the Congo [2]. |
| *Picralima nitida (Stapf) T. Durand & H. Durand (Apocynaceae)* | Contains many alkaloids [14]. |
| *Myrianthus arboreus P. Beauv. (Urticaceae)* | Three peptide alkaloids have been isolated from this plant [14]. |

References

1. dos Santos FSD, de Souza LPA, Siani AC. 2008 [Chaulmoogra oil as scientific knowledge: the construction of a treatment for leprosy]. *Historia, ciencias, saude--Manguinhos* **15**, 29–47. (doi:10.1590/S0104-59702008000100003)

2. Bouquet A. 1972 *Plantes médicinales du Congo-Brazzaville: Uvariopsis, Pauridiantha, Diospyros, etc.* . See https://books.google.es/books/about/Plantes_m%C3%A9dicinales_du_Congo_Brazzavill.html?id=RcQ9AAAAMAAJ&redir_esc=y.

3. OUA/STRC. 1985 RAPPORT D’ACTIVITE DE L’OUA/CSTR 1984-85.

4. Kambu K, Tona L, Luki N, Cimaga K, Makuba W. 1989 Evaluation de l’activité antimicrobienne de quelques préparations traditionnelles antidiarrhéiques utilisées dans la ville de Kinshasa-Zaïre. *Bull. Méd. Trad. Pharm.* **3**, 15–24.

5. Salatino A, Faria Salatino ML, Negri G. 2007 Traditional uses, Chemistry and Pharmacology of Croton species (Euphorbiaceae). *J. Braz. Chem. Soc* **18**, 11–33.

6. Plenderleith K. 1997 RICINODENDRON HEUDELOTII A State of. Knowledge Study undertaken for the Central African Regional Program for the Environment, University of Oxford.

7. Séverin Elisée T *et al.* 2020 Acute and Subacute Toxicity Studies of the Combination of the Aqueous Extracts of Trunk Bark of Musanga cecropioides R. Br. (Cecropiaceae) and Fruits of Picralima nitida (Stapf) T. Durand & H. Durand (Apocynaceae). *Saudi Journal of Medical and Pharmaceutical Sciences Abbreviated Key Title: Saudi J Med Pharm Sci ISSN* (doi:10.36348/sjmps.2020.v06i04.002)

8. Akaniro-Ejim NE, Ubani CS, Nubila NI, Nzei AA, Nwodo UU, Okoh AI. 2016 Evaluation of Saponin Extract from Vitex doniana and Pentaclethra macrophylla for Antibacterial Activity. (doi:10.3390/app6060180)

9. Heng L, Vincken JP, van Koningsveld G, Legger A, Gruppen H, van Boekel T, Roozen J, Voragen F. 2006 Bitterness of saponins and their content in dry peas. *Journal of the Science of Food and Agriculture* **86**, 1225–1231. (doi:10.1002/JSFA.2473)

10. Kambu K.;, Cimanga Kanianga;, Kikweta C.;, Matondo Nsangu B;, Nlandu M. 2009 Pharmacopée Traditionnelle de la République démocratique du Congo. *Ministry of Health DRC*.

11. Okagu IU, Ndefo JC, Aham EC, Udenigwe CC. 2021 Zanthoxylum Species: A Comprehensive Review of Traditional Uses, Phytochemistry, Pharmacological and Nutraceutical Applications. *Molecules* **26**. (doi:10.3390/MOLECULES26134023)

12. Demel Adou, A.; Mida Kabran, G.R.; N’guessan, A.H.O.; Kablan, A.L.C.; Mamyrbekova-Békro, J.A.; & Békro Y. 2019 Analyse phytochimique d’un extrait coumarinique de feuilles de Zanthoxylum gilletii de Côte d’Ivoire. *J. Soc. Ouest-Afr. Chim.* **47**, 26–31.

13. Dounias E. 2008 Protabase Record display. In *Plant Resources of Tropical Africa - Medicinal plants* (eds GH Schmelzer, G-F A.), pp. 1–10. Backhuys Publishers.

14. Bouquet A, Debray M. 1974 Plantes médicinales de la Côte d’Ivoir. *Travaux et Documents de l’ORSTOM,* **32**, 232.

15. Nissim I, Dagan-Wiener A, Niv MY. In press. The Taste of Toxicity: A Quantitative Analysis of Bitter and Toxic Molecules. (doi:10.1002/iub.1694)
